# Evaluation of Nisin-Loaded PLGA Nanoparticles Prepared with Rhamnolipid Cosurfactant against *S. aureus* Biofilms

**DOI:** 10.3390/pharmaceutics14122756

**Published:** 2022-12-09

**Authors:** Ayşe Üstün, Serkan Örtücü

**Affiliations:** Department of Molecular Biology and Genetics, Faculty of Science, Erzurum Technical University, Erzurum 25050, Turkey

**Keywords:** PLGA nanoparticles, rhamnolipid, *Staphylococcus aureus*, biofilm

## Abstract

In this article, nisin(N)-loaded poly lactic-co-glycolic acid (PLGA) nanoparticles (NPs) were prepared using the single-solvent evaporation method with a rhamnolipid(R) cosurfactant. The antibacterial–antibiofilm effects of the prepared formulation and free nisin were evaluated against *S. aureus* (ATCC 25923). The characterization of NPs was analyzed using scanning electron microscopy (SEM), Zetasizer and Fourier-transform infrared spectroscopy (FTIR). The drug encapsulation efficiency and loading capacity percentages of NPs were calculated by the spectrophotometric method. The drug release of N-loaded PVA-R-PLGA NPs was determined by the dialysis bag method. The antibacterial and antibiofilm activity of N-PVA-R-PLGA NPs was determined. PVA-R-PLGA-NPs were found to be spherical with sizes of ~140 nm, according to the SEM analysis and surface charge of N-PVA-R-PLGA NPs −53.23 ± 0.42 mV. The sustained release of N (≥72% after 6 h) was measured in PVA-R-PLGA-NPs. The encapsulation efficiency percentage of N-PVA-R-PLGA NP was 78%. The MIC values of free nisin and N-PVA-R-PLGA NPs were 256 μg/mL and 64 μg/mL, respectively. The antibiofilm inhibition percentages of free nisin and N-PVA-R-PLGA NPs were 28% and 72%, respectively. These results reveal that N-PVA-R-PLGA NPs are a promising formulation for use in infections caused by *S. aureus* compared to free nisin.

## 1. Introduction

*Staphylococcus aureus (S. aureus)* is considered as a pathogen that threatens human health by the World Health Organization. *S. aureus* attaches to medical implants and tissues. After this attachment, bacterial cells form a polymer-based matrix layer, forming structures called biofilms [1,2]. Previous studies have reported that biofilms show resistance to antimicrobials due to altered metabolism, substrate accessibility, permeability and EPS formation [3]. To combat bacterial infections in the pharmaceutical industry due to problems such as antibiotic resistance, low activity of antimicrobials in cells, toxicity to healthy tissues, and poor biopharmaceutical properties encountered in current treatment approaches, researchers have proposed new nanobiotechnology-guided drug delivery systems. Nanoparticles are seen as a promising source for drug delivery systems due to their small size and physicochemical properties suitable for modification and biocompatibility [4]. Also, using biodegradable materials to prepare nanoparticles allows for sustained drug release over days or even weeks within the target site, increasing the therapeutic benefit. These different properties make nanoparticles ideal drug delivery systems for managing severe diseases such as intracellular infections or cancers, thus overcoming some of the limitations of conventional therapeutics. In this study, polymeric drug delivery systems were preferred due to their biodegradability, non-toxicity and physicochemical properties that can be controlled by production variables. PLGA (poly lactic-co-glycolic acid), which has a polymeric structure, is preferred for controlled-release drug delivery systems due to its biocompatibility, biodegradability, and mechanical strength and continues to be used to develop new controlled-release systems [5,6,7]. According to the study of Haider et al. (2022), the release of nisin from PLGA NPs was observed at a cumulative rate of 85.78% ± 2.07 for 72 h [8]. According to the study of Antonov et al. (2022), continuous release of the antibiotic was achieved by using an “organic solvent-free” approach using polylactic-co-glycolic acid (PLGA) microparticles containing levofloxacin. The bactericidal efficacy of the established drug delivery system has been investigated both in vitro and in vivo, and a promising candidate as controlled release formulations for anti-tuberculosis has been found [9]. These results clearly show that PLGA-based drug delivery systems have been the right choice for controlled release.

The drug encapsulated in our study is the nisin protein, an antimicrobial polypeptide (AMP). Nisin, a bacteriocin, is a cationic and amphiphilic inexpensive AMP produced by *Lactococcus lactis* strains. This antimicrobial polypeptide consists of 34 amino acids and has a molecular weight of 3.5 kDa. Nisin causes the effective inhibition of *Bacillus* and *Clostridium* spores as well as a broad spectrum of Gram-positive bacteria (*Staphylococcus* and *Listeria*) [10]. Nisin is widely used in the food industry due to its stable nature as well as clinical im-portance, but various factors such as salt concentration in the food system, divalent ions, pH of the food matrix, glutathione and proteolytic enzymes limit its use. These factors revealed the necessity of using different formulations in addition to free nisin in order to protect and increase nisin activity [11]. In a study, the antimicrobial activities of nisin against *Listeria* strains encapsulated in phosphatidylcholine (PC) liposomes containing chitosan or chondroitin sulfate by thin-film hydration method were evaluated. It was determined that encapsulated nisin has 21% more efficacy in inhibiting *Listeria monocytogenes* than nisin alone [12]. According to another study by Correia et al. (2015), the antimicrobial activity of nisin protein combined with PLGA matrix against *Lactobacillus sakei* was determined under different conditions and evaluated in terms of drug release profile. It showed a strong inhibitory activity by giving a value of 2.70 log CFU/mL at pH 4.5 and showed sustained release activity for 2 weeks in an 8-month drug release profile [13]. In a study by Niaz et al. (2019), rhamnosomes (RS) were developed as a result of combining the membrane of nano-liposomes with rhamnolipids (RL). Self-active rhamnosome nano-vesicles (RSNVs), which were loaded with bacteriocin (nisin Z) to achieve broad-spectrum antimicrobial activity and had rhamnolipids incorporated into the lipid bilayer, exhibited an increase in nisin encapsulation efficiency from 47 ± 4% to 88 ± 7% [11]. The addition of rhamnolipids to nisin-loaded liposomes resulted in the twofold inhibition of antibiofilm. Thus, this study supports the idea that the nisin-rhamnolipid interaction increases the encapsulation percentage and is a potential source for drug delivery systems to be used for antimicrobial purposes. There is no previous research using PLGA NPs with a PVA-R surfactant approach.

We aim to investigate the effect of PLGA NPs for nisin, prepared using rhamnolipids, which have demonstrated their effective properties in our previous study [14]. Nisin-loaded PLGA NPs were prepared by solvent evaporation method and evaluated in terms of zeta potential, size, percent encapsulation and antimicrobial–antibiofilm activity.

## 2. Materials and Methods

### 2.1. Materials and Microorganisms

Nisin 2.5%, PLGA (MW = 76,000–115,000), 75:25, PVA (MW = 30,000–70,000), acetone (HPLC grade), ethanol (100%) and dialysis bag (MWCO: 12 kDa) were purchased from Sigma Aldrich. Rhamnolipid (90%, pure) was purchased from AGAE Technologies. NutriCulture Phosphate-Buffered Saline (PBS) was purchased from EcoTech Biotechnology. *Staphylococcus aureus* (ATCC 25923) was used as the reference pathogen and was maintained in Mueller Hinton Agar (MHA) at 37 °C.

### 2.2. Preparation of NPs (PLGA-NPs)

Before preparing the drug delivery system, nisin (2.5%) was purified by ethanol extraction. A total of 200 mg of commercially purchased nisin (2.5%) was weighed and suspended in 10 mL of 50% ethanol. The suspension was then stirred at room temperature for 8 h and then centrifuged at 1.520 g for 5 min. After centrifugation, the supernatant was lyophilized. The nisin protein obtained after lyophilization was dissolved with 0.02 N HCL and used in the drug delivery system [15]. The drug delivery system was designed with PVA-rhamnolipid (1:1). The formulation was prepared according to the previous study with some modifications [14]. Briefly, 2.5 mg of purified nisin was used as a drug, and acetone was used as an organic solvent. To remove the free N from NP preparation, the nanoformulation was immersed in 5 mL of distilled water in a dialysis bag and dialyzed for 1 h (MWCO: 12 kDa). Dialysis was carried out at room temperature at 200 rpm on a magnetic stirrer. This assay, which will be used to remove the free drug, has been preferred because it provides advantages over other methods (ultrafiltration, ultracentrifugation) in terms of sensitivity and accuracy. PLGA nanoparticles free nisin were also prepared for negative control. The prepared formulation was used freshly in future experiments [16].

### 2.3. Characterization of NPs (PLGA-NPs)

#### 2.3.1. Mean Particle Size, Zeta Potential and Polydispersity Index

A zeta sizer Nano ZS (Malvern Instruments, Malvern, UK) was used for the determination of Mean particle size (d.nm), polydispersity index (PDI) and zeta-potential values NPs. In brief, the measurements were determined by the dynamic light scattering method at 20 °C, and samples for analysis were prepared with appropriate dilutions.

#### 2.3.2. Scanning Electron Microscopy (SEM)

For morphological characterization, NP formulations were coated with gold and examined under SEM (FEI Quanta Feg-450). The magnification of the image was set to ×20,000 [17].

#### 2.3.3. Fourier Transform Infrared (FTIR) Spectrometry

Infrared spectra will be obtained by using NP formulations and the nisin protein FT-IR to determine the polymer-active substance interactions and to detect the drug presence in the prepared drug delivery formulations. The IR (infrared) spectra of the samples will be acquired in a reflectance (ATR) mode and the spectral region of 400 to 4000 cm^−^^1^ [18].

#### 2.3.4. Encapsulation Efficiency (EE), Drug Loading Capacity (DL)

The solution in the release medium after dialysis of excess nisin from the NPs was measured to determine the EE% and DL% of the NPS. Then, the solution was measured with Multiskan™ GO Microplate Spectrophotometer at 215 nm, and the amount of N was quantified using an N calibration curve. EE% and DL% were measured using the following equations [19,20]. The EE% determination method is an indirect method since the amount of encapsulated nisin is measured from the suspension released from the dialysis bag and subtracted from the initial amount of nisin. The NP formulation in the ‘total amount of formulation’ is the initial theoretical amount.

EE% = (amount of N loaded in NP)/(total amount of N used) × 100

DL% = (amount of N loaded in NP)/(total amount of formulation) × 100

### 2.4. In Vitro Nisin Release

In vitro release test of Üstün and Örtücü (2022) was carried out by making some changes to the protocol according to the dialysis bag technique [14]. For this technique, the dialysis bag was used as MWCO:12 kDa.

NP formulation was measured spectrophotometrically at 215 nm at specific time intervals (0, 6, 12, 24, 48 and 72 h).

### 2.5. Evaluation of Antibacterial Activity

NPs’ formulations (N-PLGA-NPs and the PLGA-NPs) were dissolved in a sterile buffer solution in the same concentration. The liquid dilution method was used to measure the MIC (minimum inhibitory concentration) value to determine antibacterial activity. In summary, 100 µL of bacterial cells at 0.5 McFarland concentration and specific concentrations of substances (0.25, 0.5; 1; 2; 4; 8; 16; 32; 64; 128; 256 µg/µL) were used in 96-well plates. The total volume was made up to 200 µL. Medium containing only medium was used as a negative control, and the bacterial cell group was used as the positive control. MIC values of test samples were recorded as the lowest concentration that inhibited bacterial growth after 24 h of incubation at 37 °C [21].

### 2.6. Evaluation of Antibiofilm Activity

Antibiofilm activity of nisin and NP formulation were determined with the crystal violet (CV) assay. For the biofilm formation inhibition test, 50 µL of NP formulations (in the same concentrations and dissolved in PBS), 75 µL of MHB medium, and 75 µL of 1-day-old bacterial cells (*S. aureus*) adjusted to 0.5 McFarland were added to 96-well plates with polystyrene properties at 37 °C and incubated for 48 h. At the end of the period, all the media contents were removed, and all wells were washed three times with PBS. The wells were then treated with 0.5% crystal violet, and after washing, the biofilm mass was suspended with 30% acetic acid. The biofilm% inhibition was measured by using the following equations [22]. Untreated bacteria cells are used as control.

% inhibition = ((untreated bacterial cells−treated bacterial cells)/(untreated bacterial cells)) 595 nm × 100

### 2.7. Statistical Analysis

The results were determined as mean ± SD and analyzed for statistical significance (*p* < 0.05) by one-way ANOVA (GraphPad Software Inc. San Diego, CA, USA).

## 3. Results

### 3.1. Physicochemical Characterization of NPs

**Figure 1:**
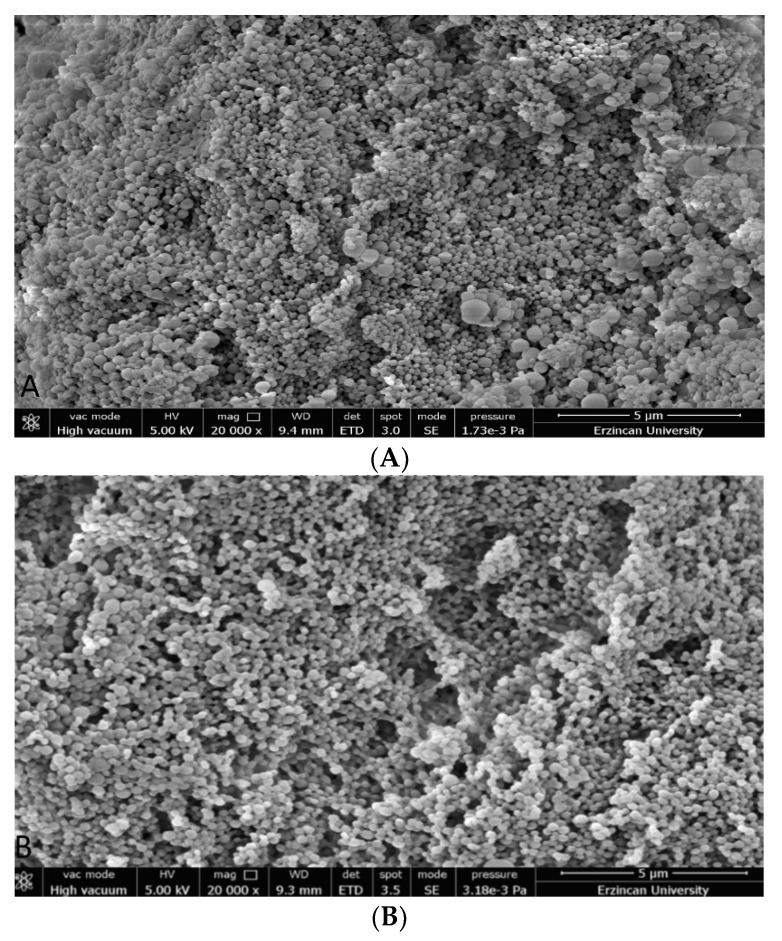
SEM image of PLGA NP, free nisin-loaded PLGA-NP (**A**), nisin-loaded PLGA-NP (**B**).

The morphology of the NP formulations was characterized by SEM. It was determined that the NP formulation prepared with PVA-R surfactant morphology was spherical and mean 140 nm (Figure 1).

The ZP, PDI and d. nm values of the NP formulation are shown in Table 1. The formulation prepared with PVA-R surfactant was compared with loaded and unloaded N.

### 3.2. Encapsulation Efficiency and Drug Loading Capacity of N

The EE% and DL% values of N-PVA-R-NPs are 78% and 25%, respectively. (Table 2).

### 3.3. Fourier Transform Infrared (FTIR) Spectrometry

**Figure 2:**
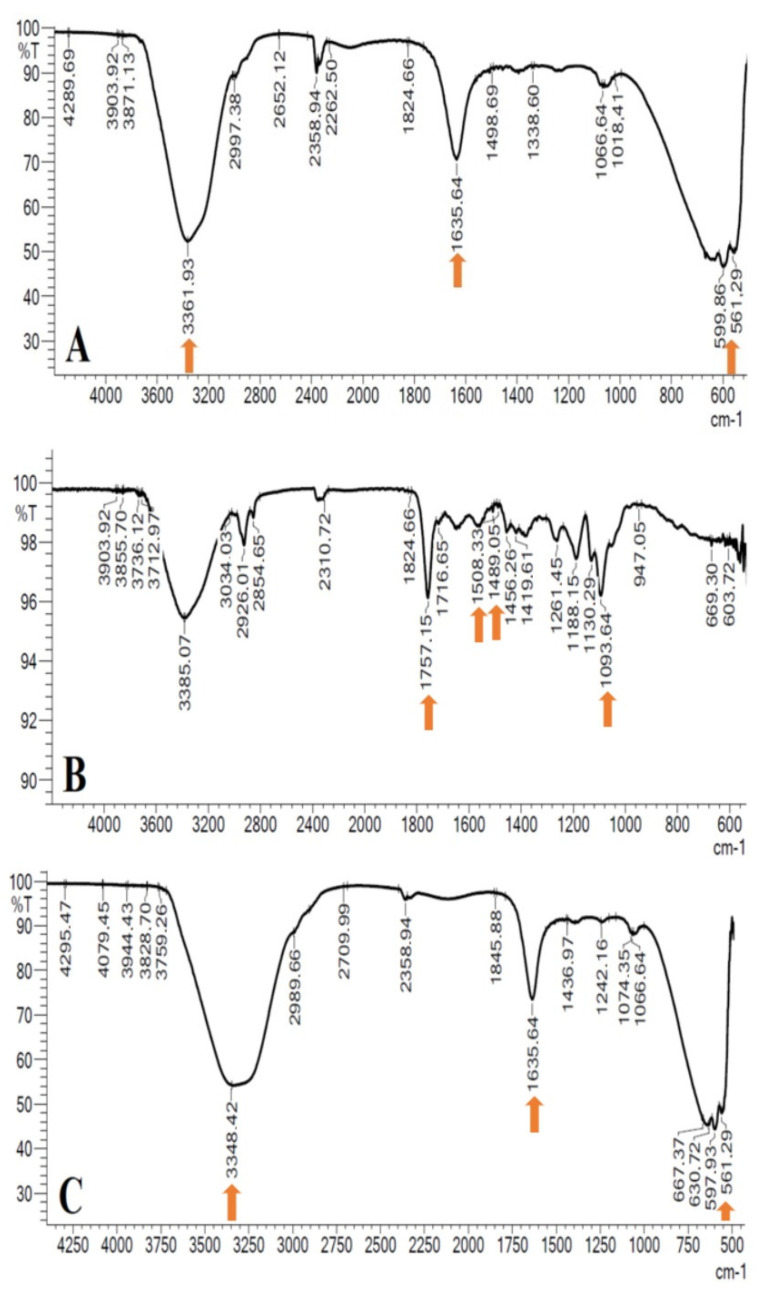
FTIR spectral comparison of nisin (**A**), free nisin-loaded PLGA NPs (**B**), nisin-loaded PLGA NPs (**C**).

Figure 2 shows the FTIR spectra of nisin, free nisin-loaded PLGA NPs and nisin-loaded PLGA NPs. The spectra of nisin give broad bands at 3361 cm^−^^1^ due to the OH stretching of the COOH group. The peak at 1635 cm^−^^1^ is related to the amide group. In the spectra of nisin-loaded PLGA NPs, the peak at 3348 cm^−^^1^ is related to the free O–H group of COOH, and the peak at 1645 cm^−^^1^ is related to the amide group. The peak at 597 cm^−^^1^ is due to the specific peak of nisin. Although the FTIR spectra of nisin-loaded PLGA NPs were consistent with the FTIR spectra of free nisin, the peaks of PLGA NPs at 1757 cm^−^^1^, 1508 cm^−^^1^, 1489 cm^−^^1^ and 1093 cm^−^^1^ were significantly reduced. The decrease or disappearance of these peaks showed an interaction between nisin and PLGA NPs and that nisin is encapsulated into PLGA NPs [21].

### 3.4. In Vitro Nisin Release

**Figure 3:**
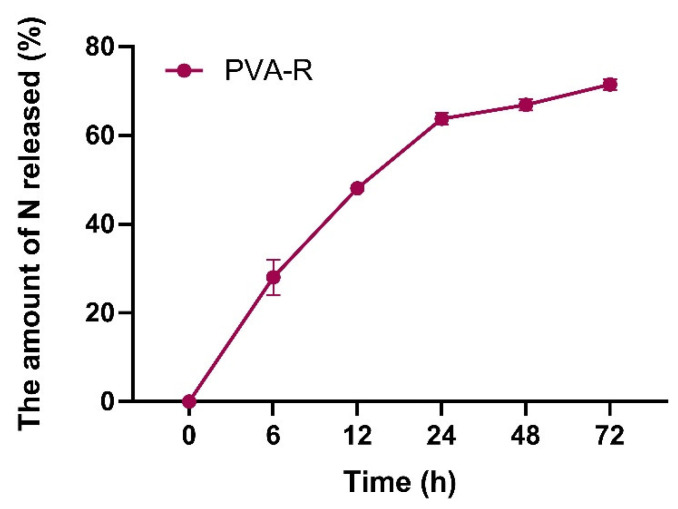
In vitro drug release profiles of N-loaded NPs (prepared with PVA-R surfactant). Results were represented as mean ± SD, *n* = 3.

Drug release profiles of N-PVA-R-PLGA NPs were evaluated with an in vitro release assay with dialysis bag assay (Figure 3). It was found that PVA-R PLGA-NPs exhibited biphasic release in PBS, with an initial burst release of about 28% after 6 h. The second phase release profile was evaluated with about 72% of the drug released for 72 h.

### 3.5. Evaluation of Antibacterial Activity

The MIC values of nisin and PVA-R-PLGA NPs against *S. aureus* were 256 μg/mL and 64 μg/mL, respectively.

### 3.6. Evaluation of Antibiofilm Activity

**Figure 4:**
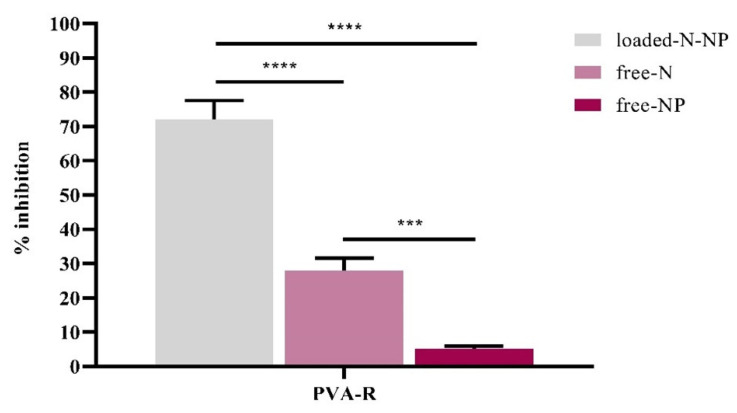
The efficiency of biofilm inhibition of PVA-R NPs. PVA- R NPs, PLGA-NP prepared with PVA-R surfactant. Results were determined as mean ± SD, *n* = 3. Statistically significant differences between groups: *** *p* < 0.0005, **** *p* < 0.0001 (one-way ANOVA).

The results of the CV assay revealed the high potential of the N-loaded PVA-R-NPs for inhibition and treatment of *S. aureus* biofilms compared to free N and NPs (Figure 4). The percentage of antibiofilm inhibition of N-loaded PVA-R-NPs and nisin was 72% and 28%, respectively. The antibiofilm percentage of blank PLGA NPs was analyzed as 5%.

## 4. Discussion

Nanobiotechnological drug delivery systems have been preferred for bioactive material encapsulation in the last decade due to their biocompatibility and bioavailability. Nisin protein has been encapsulated in nanocarrier systems to increase bioavailability and has been used in the food industry and the fight against bacterial biofilms. For this purpose, we used the PVA-R-PLGA-NP nanobiotechnological drug delivery system for nisin. Studies with rhamnolipids confirm the accuracy of this choice. Marangon et al. (2020) added rhamnolipids to chitosan NPs, causing the NPs to decrease in size and polydispersity index and show a more positive surface charge and improved stability. It was determined that the formulation prepared with the combination of rhamnolipids showed more effect against *S. aureus* and *S. epidermidis* biofilms compared to chitosan NPs [23]. In the study of Falakaflaki et al. (2022), nanocomposites with antimicrobial properties were made by encapsulating bone-regenerative substances with rhamnolipids. The prepared nanocomposites showed strong antimicrobial activity against *S. aureus* with an antibiofilm activity of 43.76% ± 1.65%. An increase in genes related to bone regeneration was detected [24]. In the study of Cheow et al. (2012), a lipid–polymer combination was used against Pseudomonas aeruginosa biofilm, and rhamnolipids were used as a triggering agent for the release of antibiotics from the drug delivery system [25].

N-PVA-R-PLGA NPs had spherical morphology and zeta potential suitable for the nanobiotechnological drug delivery system in our study. The negative charge of NPs may be due to the surface charge of PLGA. The high zeta potential value provides stability to the nanocarrier system by preventing the aggregation of molecules. The high zeta potential of nisin-loaded PLGA NPs provides an advantage for the drug delivery system. For the homogeneous distribution of NPs, the PDI value should be between 0 and 1, and according to the results, N-PVA-R-PLGA NPs have a homogeneous distribution [26]. The size of N-PVA-R-PLGA NPs was determined to be 140 nm on average as a result of SEM analysis. The effect of surfactant addition and different surfactant concentrations on NP size has been proven. In the study of Lee et al. (2021), the formulation prepared with rhamnolipids (PLGA NPs encapsulated with doxorubicin and erlotinib) reduced the size by approximately 128 nm compared to the formulation prepared without rhamnolipids [27]. According to the study by Koppolu MS et al. (2010), they found that the size of PLGA NPs prepared with a PVA surfactant decreased as the surfactant concentration increased as a result of factorial analysis [28].

In addition, in our previous study, PLGA NPs prepared with a PVA-R surfactant, compared to PLGA nanoparticles prepared only with a PVA surfactant, showed superior properties in terms of size, zeta potential and PDI [14].

Nisin release from PLGA NPs showed biphasic release, burst phase and the second phase (Figure 3). An in vitro release study of N-PLGA-NPs was performed in PBS buffer at pH = 7.4. The release kinetics of NPs were studied for 72 h. Although the release of the drug is a combination of polymer degradation and diffusion, a burst release of 28% occurred during the first 6 h due to the short diffusion paths of molecules held close to the surface and molecules adsorbed to the surface. The regular release after 6 h in our study is a desirable situation in drug delivery studies.

According to MIC values, N-PVA-R-PLGA NP was effective in reducing the bacterial viability of *S. aureus* compared to free N. (four times more). These results were found to contain similar results to those reported by da Silva et al. (2014) and Üstün and Örtücü (2022) [12,14]. They presented that the antimicrobial properties of bioactive agents loaded PLGA nanoparticles are better than free formulations. Similarly, Arasoglu et al. (2015) showed that caffeic acid phenethyl ester encapsulated in PLGA showed higher antimicrobial activity than their non-encapsulated forms [21].

Incorporating AMP of different natures (organic, inorganic or hybrid) into nanobiotech drug delivery systems represents an effective therapeutic strategy to combat biofilms. NPs can be developed to increase penetration into cells and selectively target or release drugs. Moreover, the use of NPs as carriers allows an increase in drug efficacy by overcoming resistance mechanisms in bacteria. The antibiofilm inhibition percentage of N-PVA-R- PLGA NP was 72% (Figure 4).

The antibiofilm inhibition percentage of nisin is 28%, and the difference (significant difference (**** *p* < 0.0001)) shows that nisin encapsulation increases the antibiofilm efficiency approximately 2.5 times. According to the study of Niaz et al. (2019), rhamnosomes were made by combining nano-liposomes with rhamnolipids. An approximately 80% reduction in biofilm biomass was observed by loading nisin into rhamnosomes. In this study, the nisin protein was loaded with rhamnosome nano-vesicles, and its antibiofilm properties were investigated after treatment with crystal violet assay. According to the results, it was determined that the drug delivery system prepared with rhamnolipids reduced the attachment of biofilm cells by reducing the tension between the biofilm cells and the surface [11].

In one study, an NP formulation containing rhamnolipids and phospholipids was used to encapsulate amoxicillin and pectin sulfate, an anti-adhesive agent. This study demonstrated that the nanocarrier could significantly disrupt the *Helicobacter pylori* biofilm by eliminating EPS and also reducing both the adhesion and colonization of bacteria [29].

In our previous study, we benefited from the slow release of the active ingredients of PLGA and the advantages of rhamnolipids. According to these studies, rhamnolipids appear as encapsulation agents or cosurfactants. The nisin delivery performed in this study with PLGA NPs may result in the development of a new therapeutic system.

## 5. Conclusions

Biofilms originating from *S. aureus* threaten human health, and researchers have turned to nanobiotechnology for this in recent years. The use of nanobiotechnological drug delivery systems to increase the effectiveness of antimicrobial agents is very promising. For this purpose, PLGA NPs prepared with a PVA-R cosurfactant were used in our study and examined in terms of zeta potential, intermolecular interactions, PDI, size, in vitro release, drug encapsulation efficiency and antibiofilm activity. As a result, we found that nisin-loaded PLGA NPs are a more suitable drug delivery system compared to nisin alone.

## Figures and Tables

**Table 1:** Zeta potential, polydispersity index and mean particle size of NP formulations (*n* = 3) *.

Formulations	ZP ± SD	PDI ± SD	d.nm ± SD
**PVA-R-NP**	−33.5 ± 1.56 mV	0.497 ± 0.042	374.2 ± 21
**N-PVA-R-NP**	−53.23 ± 0.42 mV	0.339 ± 0.013	371 ± 1.13

Note: Results were determined as mean ± SD, *n* = 3. * ZP, zeta potential; PDI, polydispersity index; d. nm, mean size; PVA-R-NP, NPs prepared with PVA-R surfactant; N-PVA-R-NP, nisin-loaded NPs prepared with PVA-R surfactant.

**Table 2:** EE% and DL% value of N-PVA-R-NP *.

	EE% ± SD	DL% ± SD
**N-PVA-R-NP**	78 ± 3.42	25 ± 2.3

Note: Results were determined as mean ± SD, *n* = 3. * EE, Encapsulation efficiency; DL, drug loading capacity; N-PVA-R-NP, nisin-loaded NPs prepared with PVA-R surfactant.

## Data Availability

Not applicable.
